# Talimogene Laherparepvec in Advanced Mucosal Melanoma of the Urethra Upon Primary Resistance on Immune Checkpoint Inhibition: A Case Report

**DOI:** 10.3389/fonc.2020.00611

**Published:** 2020-05-08

**Authors:** Anne Fröhlich, Friederike Hoffmann, Dennis Niebel, Eva Egger, Guido M. Kukuk, Marieta Toma, Judith Sirokay, Thomas Bieber, Jennifer Landsberg

**Affiliations:** ^1^Department of Dermatology and Allergy, Rheinische-Friedrich-Wilhelms-Universität Bonn, Bonn, Germany; ^2^Department of Gynaecology, Rheinische-Friedrich-Wilhelms-Universität Bonn, Bonn, Germany; ^3^Department of Radiology, Kantonsspital Graubünden, Chur, Switzerland; ^4^Department of Pathology University Hospital Bonn, Rheinische-Friedrich-Wilhelms -Universität Bonn, Bonn, Germany

**Keywords:** immune checkpoint blockade, intralesional treatment, mucosal melanoma, primary resistance, talimogene laherparepvec

## Abstract

**Background:** Mucosal melanomas including melanomas of the urogenital tract represent a rare type of melanoma characterized by low mutational burden and poor prognosis. Immune checkpoint inhibition has so far only been assessed in a limited number of mucosal melanoma patients and, in contrast to response in cutaneous melanoma, was associated with disappointing response rates. The oncolytic viral immunotherapy Talimogene laherparepvec (T-VEC) has recently been approved for treatment of locally advanced or unresectable melanoma. T-VEC combines direct oncolytic effects with local and systemic immune-mediated anti-tumor response. Our rationale to use T-VEC in this case was an expected augmentation of immunogenicity by tumor lysis to overcome primary resistance of a mucosal melanoma to immune checkpoint blockade.

**Objective:** To report the first case of an advanced mucosal melanoma of the urethra treated with intralesional application of Talimogene laherparepvec.

**Case Report:** A 78-years old female patient was diagnosed with an advanced mucosal melanoma of the urethra with inguinal lymph node metastases and intravaginal mucosal metastases. Shortly after surgical resection of the tumor mass, intravaginal mucosal metastases, and new nodal metastases in proximity of the left iliac vessels were diagnosed. The patient was treated with the anti-PD1 antibody pembrolizumab and obtained a stable disease lasting for 30 weeks. However, upon checkpoint inhibition the patient developed a loco-regional progressive disease featuring bleeding intravaginal metastases, while nodal metastases remained stable. We stopped treatment with pembrolizumab and administered T-VEC directly into the intravaginal mucosal metastases. After five injections T-VEC yielded a partial response with clinical regression of the injected mucosal metastases. Disease remained stable for 16 weeks under biweekly T-VEC treatment. Thereafter the patient showed disease progression in nodal metastases. T-VEC was discontinued. Immunotherapy with pembrolizumab was restarted but failed to achieve a response. Finally, targeted therapy with imatinib was induced in presence of a druggable *c-KIT* mutation, leading to a considerable response of all tumor sites that is still ongoing.

**Conclusion:** T-VEC represents an effective and well-tolerated treatment option for patients with loco-regionally advanced mucosal melanoma. In combination with immunotherapy, T-VEC bears the potential of synergistic effects to overcome the specific primary resistance of mucosal melanoma to immune checkpoint blockade.

## Background

Mucosal melanomas including melanomas located in the urogenital tract represent a rare phenotype. Primary melanomas of the urethra make up <1% of all melanomas and 4% of urethral cancers (1). Melanomas of the urogenital tract form a group of aggressive malignancies and due to hidden anatomical localization the diagnosis is often delayed, leading to poor prognosis (2). The approval of the immune checkpoint inhibitors anti-CTLA4 and anti-PD1 as well as the targeted therapy against BRAF and MEK have remarkably improved overall survival for patients with cutaneous melanoma. Since mucosal melanoma is quite uncommon, the efficacy of these therapeutic options has only been assessed in a limited series of patients and data from clinical trials is scarce. Preliminary reports suggest lower response rates of mucosal melanoma to immunotherapy compared to cutaneous melanoma, the reasons being unclear. Mucosal melanoma is characterized by a higher number of chromosomal structural aberrations and a lower mutational burden than cutaneous melanoma (3). Accordingly, mutations in *BRAF* and *NRAS* are less prevalent in mucosal melanoma, targeted therapy is only available for a small subset of patients. Some mucosal melanoma harbor *c-KIT* mutations targetable by imatinib or nilotinib (4).

Tumor infiltrating lymphocytes can be detected less frequently in mucosal melanoma than in cutaneous melanoma (5). Therefore, it has been hypothesized that mucosal melanomas tend to be less immunogenic and are consequently often primarily resistant to immune checkpoint blockade.

In patients with locally advanced or unresectable cutaneous melanoma the oncolytic viral immunotherapy Talimogene laherparepvec (T-VEC) represents an additional therapeutic option. Approval was granted by FDA and EMA in 2016 for the local injection in cutaneous, subcutaneous and nodal metastases in unresectable stage IIIB-IVM1a melanoma patients. T-VEC is a genetically modified herpes simplex virus type 1 combining direct oncolytic effects with local and systemic, immune-mediated anti-tumor response (6, 7). The phase III trial (OPTiM) which led to approval of T-VEC demonstrated an overall response rate of 26,4 %, including 10.8% complete responses (8). Patients with mucosal melanoma were excluded from the trial. To our knowledge there is no published data about intralesional treatment of mucosal melanoma or mucosal metastases with T-VEC so far. Here we report the case of a patient with intravaginal metastases of a melanoma of the urethra responding to intralesional treatment with T-VEC.

## Case Report

A 78-years old female patient was diagnosed with a mucosal melanoma of the urethra (patient characteristics: see Table 1).

**Table 1:** Medical history, clinical, histological, and molecular characteristic of the patient.

**History of primary diagnosis and medical history**	
Gender, age	Female, 78 years
Staging of primary and lymph node status	Mucosal melanoma of the urethra; Tumor thickness 10 mm (Breslow); Ulceration UN; LN (4/8 ece-); pT4, N3c, M1a, stage IV (AJCC 2017);
Mutational profile	*BRAF* wt *NRAS* wt *KIT* mutation exon 11, c.1672_1674dup p.K558dup
Adjuvant therapy	None
Medical history	Hysterectomy due to myomas Arterial hypertonia Hypercholesterolemia
Family history	Negative family history of melanoma
Psychosocial history	Widowed, 2 children and grandchildren

*UN, unknown; LN, lymph node status; ece, extracapsular lymph node extension; wt, wild type*.

**Figure 1:**
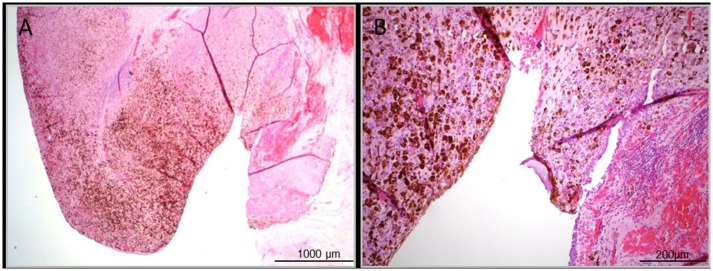
Representative histopathologic images of the ulcerated primary pigmented mucosal melanoma of the Urethra. **(A)** Overview image of the histopathologic sample, H&E stained in 2,5-fold magnification. **(B)** Detailed view, H&E stained in 10-fold magnification.

**Figure 2:**
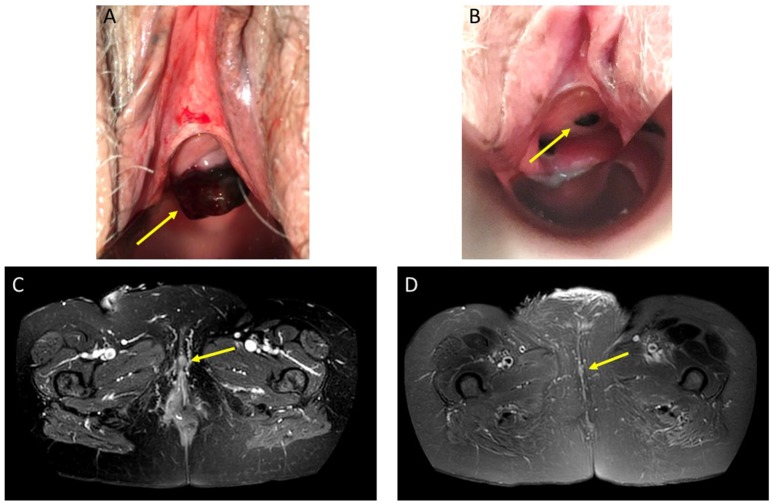
Clinical and MR image of intravaginal mucosal metastasis of a malignant melanoma of the urethra. A/B: Visual appraisal with speculum. **(A)** Target lesion before injection of T-VEC. Baseline image shows a pigmented ulcerated mucosal tumor (arrow). **(B)** Target lesion after seven injections of T-VEC: partial response with substantial regression of the injected mucosal metastasis (arrow) and cessation of intravaginal bleeding. C/D MR image. **(C)** Transverse T2-weighted fat suppressed MR image shows labial metastasis (arrow) before injection of T-VEC. **(D)** Transverse T2-weighted fat suppressed MR image 2 months upon T-VEC therapy shows complete disappearance of labial metastasis (arrow indicating the original location).

**Figure 3:**
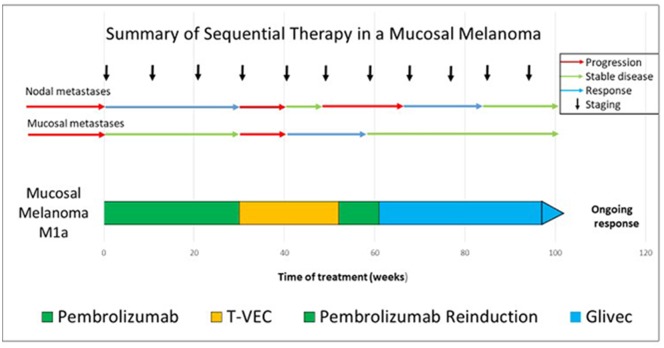
Summary of the sequential treatment in our case of advanced mucosal melanoma.

At the time of primary diagnosis, inguinal lymph node metastases were detected. A complete resection of the urethra and a radical dissection of the left inguinal lymph nodes was performed concomitantly in our surgical department. Only 1 month after the intervention intravaginal mucosal metastases were diagnosed and histologically confirmed (Figure 1). Computed tomography (CT) scans showed nodal metastases in proximity of the left iliac vessels with no option to obtain a complete resection of the tumor masses. Molecular analyses of the tumor showed wild types in the *BRAF*- and *NRAS* gene and a p.K558dup mutation of *c-KIT* on exon 11. In view of the locally advanced, inoperable melanoma a systemic therapy with the PD-1 inhibitor pembrolizumab was induced and temporary obtained stable disease. After administration of 10 cycles of pembrolizumab the patient started to suffer from recurrent vaginal bleeding, which significantly restricted the patient's quality of life. Clinical examinations revealed ulcerated pigmented intravaginal metastases. Imaging confirmed loco-regional progress without distant metastases (Figure 2). Hence, 4 weeks after the last dose anti-PD1 antibody and in agreement with our patient, we initiated treatment with the oncolytic virus T-VEC (first administration 10^6^ PFU/ml, followed by 10^8^ PFU/ml at week 3 and followed Q2W, 1–3 mL). In cooperation with our department of gynecology T-VEC was injected directly into the intravaginal mucosal metastases. The injections provoked moderate local bleeding of the mucosa, and the patient suffered from flu-like symptoms a few hours after injections. The patient did not show any signs of a herpes infection at any time. Our patient reported that the T-VEC applications were tolerable and that the side effects did not restrict her daily life. Laboratory investigations did not reveal any significant pathologic findings. After the first injections, metastases slightly seemed to increase in size, but vaginal bleeding remarkably ameliorated. After five injections T-VEC yielded a partial response with substantial regression of the injected mucosal metastases and cessation of intravaginal bleedings (Figure 2). Overall nine cycles of T-VEC were administered. The uninjected iliac lymph node metastases did not respond to oncolytic virus therapy. CT scans revealed a progression of the left inguinal nodal metastases and development of retroperitoneal nodal metastases after 16 weeks upon T-VEC. Consequently, T-VEC was discontinued and immunotherapy with pembrolizumab (2 mg/kg Q3W) was restarted. After three cycles pembrolizumab, para-aortic and mesenteric nodal metastases were diagnosed and there was a progression of the iliac and inguinal nodal metastases. The intravaginal metastases remained stable. A combined immunotherapy with PD-1- and CTLA-4-inhibition was refused by the patient due to higher risk of treatment related toxicities. With regards to the druggable *c-KIT* mutation (p.K558dup in Exon 11) we started treatment with the tyrosine kinase inhibitor imatinib (400 mg once a day). Treatment with imatinib achieved a considerable regression of all metastatic tumor sites with a still ongoing response (summary of the sequential therapies: see Figure 3).

## Discussion

Here, we present the case of a 78-years old female patient with a primary pigmented melanoma of the distal urethra. Melanoma of the urinary tract are extremely rare. The distal urethra is the most common site of occurrence of melanoma in the urinary tract. It is more common in females and elderly patients (9). In contrast to increasing incidence of cutaneous melanoma, the incidence of mucosal melanoma has remained fairly stable (10) which reflects the distinct UV-independent biology of mucosal melanoma. Due to the rarity of mucosal melanoma and its anatomic location diagnosis is often delayed and patients commonly present with advanced disease. Therefore, patients with mucosal melanoma generally have a worse prognosis than patients with cutaneous melanoma with the lowest 5-years survival (11%) for women with urogenital-tract melanoma (2, 10). First-line therapeutic strategy in primary mucosal melanoma is surgery, which is recommended to be performed early and in an aggressive manner. However, primary surgery ranges from conservative surgery with wide local excision to radical surgery with urethrocystectomy and total exenteration. It has been stated that survival after radical surgery does not differ from survival after conservative surgery (2). Therefore, in our patient no urethrocystectomy or total exenteration was performed.

For unresectable or metastatic melanoma, immune checkpoint blockade with anti-CTLA4 and anti-PD1 and targeted therapy against BRAF and MEK improved the overall survival for cutaneous melanoma. The efficacy of anti-CTLA-4 and anti-PD1 antibodies has not been specifically evaluated in larger cohorts of patients with mucosal melanoma. Yet, response rates seem to be lower than in cutaneous melanoma. A recent pooled analysis evaluated PD-1 blockade alone (86 patients) or in combination with ipilimumab (35 patients) in mucosal melanoma patients (11). Response rate for anti-PD1 monotherapy was 23.3% with a progression-free survival (PFS) of 3.0 months. For combination of nivolumab with ipilimumab response rate was 37.1% with a PFS of 5.9 months. This identifies patients who suffer from metastatic mucosal melanoma as high medical need subgroup as corresponding response rates in cutaneous melanoma were 40.9% for monotherapy and 60.4% for the combined immunotherapy with a PFS of 6.2 months in monotherapy and 11.7 months in patients treated with combined immunotherapy. Another recent retrospective study including 35 patients with mucosal melanoma produced comparable PFS of 3.9 months and a median overall survival of 12.4 months (12). The lower response rate of mucosal melanoma in comparison to cutaneous melanoma might be explained by the different genomic landscape of mucosal melanoma. Whole genome sequencing data from mucosal melanoma demonstrated a low single nucleotide mutation burden without any evidence of UV signature, but numerous large-scale copy number changes and whole chromosome gains and losses (3). A high somatic tumor mutational burden is associated with improved survival in patients receiving immune checkpoint blockade across a wide variety of cancer, including melanoma (13). Beyond, density of tumor infiltrating lymphocytes is decreased in mucosal compared to cutaneous melanoma (5), supporting the hypothesis that mucosal melanoma are less immunogenic and consequently frequently primarily resistant to immune checkpoint blockade. Our patient initially obtained a stable disease which lasted for 30 weeks. However, upon anti-PD1 therapy our patient developed a loco-regional resistance with disease progression.

The disappointing results of immune checkpoint blockade in mucosal melanoma demonstrate the need for alternative or additional treatment strategies preferentially enhancing immunogenicity of mucosal melanoma. In vulvovaginal mucosal melanoma radiotherapy has been approved to be appropriate in the (neo-)adjuvant setting (14). Beyond, combined radiotherapy and checkpoint inhibition bear the potential to create a synergistic anti-tumor response. We found one retrospective case series investigating on the so called abscopal effect in mucosal melanoma of the lower genital tract including four patients treated with combined ipilimumab and radiotherapy. In three of the four patients this therapy was followed by surgery. The study showed favorable responses suggesting further trials to follow up on this observation (15).

The vaginal mucosa constitutes a tissue with distinct inflammatory and tolerogenic properties, which are tailored to the physiologic functions as barrier tissue on the one hand and tolerance to fetal antigens in pregnancy on the other hand (16). In an experimental model, vaginal antigen exposure was followed by mucosa induced tolerance (17). However, these specific tolerogenic qualities have not been demonstrated to render the vaginal mucosa more susceptible to malignancies. The latter phenomenon has been suggested in organs with limited regenerative capacity such as the eye or the brain, a mechanism for reducing the risk of immune-mediated inflammation, also referred to as “immune privilege” (18). A recent study showed that vaginal type-II mucosa itself is an inductive site for primary CD8^+^ memory T-cells (19). In a mouse model the authors demonstrated vaginal antigen-specific CD8^+^ T-cell immune responses in the absence of lymph node involvement. The ability of vaginal mucosa to induce local immunity supports the rational for a local immunotherapy like T-VEC. During treatment with T-VEC our patient initially showed an increase of size of the intravaginal, mucosal metastases. We interpreted the initial growth of the metastases as a pseudoprogression, caused by reactive infiltration of immune cells. Median time to response among the 78 responding patients in the OPTiM approval trial was 4.1 months (range, 1.2 to 16.7 months) in the T-VEC arm. In more than half of the responding patients a pseudoprogression with appearance of new lesions or 25% initial increase of metastatic lesions was observed before response to T-VEC was achieved (8). The phenomenon of pseudoprogression has also been described in immunotherapies including anti-PD-1 antibodies and the CTLA-4 inhibitor Ipilimumab and remains a challenging task for radiologic work up and treatment evaluation (20, 21).

Several recent clinical studies have focused on treatment with T-VEC alone and in combination with other systemic treatments, specifically immunotherapy. These trials excluded patients with mucosal melanoma (8, 22–25). The published data support the idea that combining checkpoint inhibitors with oncolytic therapy may provide a synergistic efficacy by priming the tumor microenvironment (23). In our case the sequentially initiated therapies with anti-PD1 antibodies, T-VEC and re-induction of anti-PD1 antibodies occurred consecutively. Our patient did not respond to anti-PD1 therapy after T-VEC therapy systemically but showed stable mucosal melanoma metastases. It can be assumed that treatment with T-VEC might help to overcome locally acquired resistance, which is in line with the approval of the oncolytic therapy.

Targeted therapy is only suitable for a small subset of patients with mucosal melanoma, as *BRAF* or *NRAS* mutations are less common than in cutaneous melanoma. Nevertheless, 14% to 39% of mucosal melanomas harbor mutations of the *KIT* gene, which are only rarely observed in cutaneous melanomas (26, 27). In our patient the *KIT* mutation p.K558dup in Exon 11 was detected via molecular pathologic analysis. This mutation is targetable by imatinib, a tyrosine kinase inhibitor which has shown efficacy in melanoma harboring *KIT* gene mutations in exon 11 and 13. Our patient showed a good response to imatinib after intensive pretreatment with anti-PD1 and T-VEC therapy. Successful treatment with imatinib after resistance to immunotherapy has recently been shown in a patient suffering from vaginal melanoma with *KIT* p.Val559Gly mutation (4). We are aware of the limitation of this work being a single case report, which does not allow to draw general conclusions. To what extent T-VEC or targeted therapy can induce an immune response that may help to overcome primary resistance to immune checkpoint blockade should therefore be investigated in clinical trials for the rare subtype of mucosal melanoma. Recruiting a sufficient number of individuals suffering from mucosal melanoma for a clinical trial is presumed to be time-consuming. This emphasizes the great necessity of publication of case-series and case reports about this rare tumor type.

Taken together, treatment of locally advanced metastatic mucosal melanoma with T-VEC represents a therapeutic option, which should be addressed in interventional trials. Furthermore, our case underlines the rationale for the combination of T-VEC with systemic immunotherapies.

## Ethics Statement

We obtained written informed consent from the patient presented in our case report in accordance with the Helsinki Declaration of 1975. The patient consented to the treatment and the use of her data, including photos, for research, and publication.

## Author Contributions

AF and JL were involved in the study design and concept. FH, DN, EE, GK, MT, and JS were involved in data acquisition. TB revised the manuscript for critical intellectual content. All authors read and approved the final manuscript and were involved in the review and editing of the manuscript.

## Conflict of Interest

AF has received speaker's honoraria or travel expsense reimbursements from the following companies: Novartis, BMS, Almirall, and Eli Lilly Pharma. DN has received speakers' honoraria or travel expense reimbursements from the following companies: BMS, Novartis, GSK, Celgene and MSD. JS received speaker's honoraria from Novartis, BMS, MSD, and Roche. MT has received speaker's honoraria or travel expense reimbursements from the following companies: Ipsen, Roche, Astra Zeneca. JL was a consultant/advisory board member of Bristol-Myers Squibb, Merck, Novartis, and Roche. The remaining authors declare that the research was conducted in the absence of any commercial or financial relationships that could be construed as a potential conflict of interest.
